# The Impact of HIV and Parasite Single Infection and Coinfection on Telomere Length: A Systematic Review

**DOI:** 10.3390/cimb46070431

**Published:** 2024-07-08

**Authors:** Engelinah D. Macamo, Zilungile L. Mkhize-Kwitshana, Julian Mthombeni, Pragalathan Naidoo

**Affiliations:** 1 Department of Medical Microbiology, School of Laboratory Medicine and Medical Sciences, College of Health Sciences, Nelson R. Mandela Medical School Campus, University of KwaZulu-Natal, Durban 4001, South Africa; 2 Division of Research Capacity Development (RCD), South African Medical Research Council (SAMRC), Tygerberg, Cape Town 7505, South Africa; 3 Department of Biomedical Sciences, Doorfontein Campus, University of Johannesburg, Johannesburg 1710, South Africa; 4 Biomedical Sciences Department of Life and Consumer Sciences, College of Agriculture and Environmental Sciences, University of South Africa, Florida Campus, Johannesburg 1710, South Africa

**Keywords:** HIV, parasites, coinfection, telomere length shortening, biological aging

## Abstract

HIV and parasite infections accelerate biological aging, resulting in immune senescence, apoptosis and cellular damage. Telomere length is considered to be one of the most effective biomarkers of biological aging. HIV and parasite infection have been reported to shorten telomere length in the host. This systematic review aimed to highlight work that explored the influence of HIV and parasite single infections and coinfection on telomere length. Using specific keywords related to the topic of interest, an electronic search of several online databases (Google Scholar, Web of Science, Scopus, Science Direct and PubMed) was conducted to extract eligible articles. The association between HIV infection or parasite infection and telomere length and the association between HIV and parasite coinfection and telomere length were assessed independently. The studies reported were mostly conducted in the European countries. Of the 42 eligible research articles reviewed, HIV and parasite single infections were independently associated with telomere length shortening. Some studies found no association between antiretroviral therapy (ART) and telomere length shortening, while others found an association between ART and telomere length shortening. No studies reported on the association between HIV and parasite coinfection and telomere length. HIV and parasite infections independently accelerate telomere length shortening and biological aging. It is possible that coinfection with HIV and parasites may further accelerate telomere length shortening; however, this is a neglected field of research with no reported studies to date.

## 1. Introduction

Since 2000, the world’s life expectancy has increased; however, huge discrepancies remain within and across countries [1]. Sub-Saharan African countries, particularly those with poor socioeconomic status and healthcare facilities, have the lowest life expectancy rate in the world and are heavily burdened with infectious diseases [2]. Several viral (human immunodeficiency virus (HIV), hepatitis B virus, Cytomegalovirus and hepatitis C virus-2), bacterial (tuberculosis) and parasitic (malaria and soil-transmitted helminths) infections are highly endemic in sub-Saharan Africa and frequently coinfect the same host [3]. These infections can intensify oxidative stress and inflammation, leading to immune senescence, apoptosis, cellular damage and accelerated biological aging [4].

Telomere length is a hallmark of biological aging in the body [5]. A shorter telomere length may indicate that a host is biologically aging at a faster rate. Telomeres are double-stranded DNA sequences composed of the highly conserved guanine-rich hexonucleotide repeat expansion 5′-TTAGGG-3′. These nucleoprotein complexes are located at the termini of chromosomes and maintain chromosomal stability and integrity during cell division and replication [6]. The telomerase enzyme plays a role in telomere length elongation and maintenance in germ cells. Somatic cells lack telomerase activity, resulting in telomere length shortening with the progression of age [6]. Telomere length is a biomarker for biological aging and is concomitantly shortened with human aging [7]. Telomere shortening occurs in certain cell types and is linked to increased mitosis, especially during the presence of chronic infections and inflammatory conditions [8]. In response to infections, key immune cells like T cells, B cells and macrophages multiply rapidly to strengthen the body’s immune response [9]. Continued infections or inflammatory signals can cause continuous cell activation and division cycles. With each cell division, telomeres shorten due to the end-replication problem, where DNA polymerase is unable to fully replicate the ends of linear chromosomes. This process is further enhanced by pro-inflammatory cytokines such as TNF-α, IFN-γ and IL-6, which promote the growth of immune cells. Specifically, IL-6 stimulates the development and proliferation of T and B cells [10]. These powerful cytokines create a persistent inflammatory environment that spurs immune cells to undergo repeated rounds of division, resulting in telomere shortening. Infection and inflammation pose a threat to cells in certain tissues. To compensate for cell loss, the body triggers compensatory proliferation, prompting surviving cells to divide. In damaged tissues, stem and progenitor cells undergo increased mitosis to facilitate tissue healing and renewal [11]. If telomerase activity is inadequate to fully compensate for telomeric DNA loss during cell division, accelerated cell turnover may hasten telomere shortening. Replicative senescence begins when telomeres reach a critical length and continue to shrink with each successive cell division [12]. 

The shortening of telomeres resulting from natural processes (e.g., stress and cellular aging) reflects a competing and non-exclusive causal pathway linking the rate of aging and immunological responses [13]. Cumulative oxidative stress and chronic inflammation together with genetic and epigenetic modifications can trigger aberrant telomere length shortening, leading to chromosomal instability, gene expression changes, impaired cellular function and viability, biological aging and cellular senescence [14]. Senescent cells, also known as the senescence-associated secretory phenotype (SASP), stop dividing but remain metabolically active and often release inflammatory chemicals [15]. The accumulation of senescent cells accelerates telomere shortening in neighboring cells by sustaining a cycle of damage and compensatory proliferation [16]. This process leads to tissue malfunction and persistent inflammation [16]. Chronic viral infections such as HIV, hepatitis B and hepatitis C continuously activate immune cells and cause their proliferation [17]. For example, HIV infection causes T-cell turnover and prolonged immunological activation, resulting in significant telomere shortening in T cells. Prolonged immunological responses are triggered by persistent bacterial infections like tuberculosis, which promote the growth of macrophages and lymphocytes, leading to telomere attrition in these cells [18]. Depending on the species and host response, parasitic diseases like leishmaniasis can either cause chronic inflammation, which increases cell turnover and telomere shortening, or limit reactive oxygen species (ROS) generation, which promotes telomere stability [19]. 

The process of telomere shortening is influenced by a multitude of cellular and molecular processes [20]. These encompass telomerase activity, the DNA damage response, oxidative stress, chronic inflammation, the inherent constraints of DNA replication and the regulation of telomere-binding proteins. During DNA replication, the 3′ ends of linear chromosomes are not fully replicated by DNA polymerase, leaving a small single-stranded overhang [21]. With each cell division, the degradation of this overhang causes the telomeres to gradually shrink. The end-replication issue causes a cell’s telomeres to slightly shorten with each division. Following numerous divisions, telomeres attain a certain length, which triggers a DNA damage response and results in replicative senescence, a condition in which cells cease to divide but retain metabolic activity [22].

Oxidative DNA damage, particularly in the telomeres, can be brought on by ROS produced during cellular metabolism or in response to environmental stress [23]. Guanine, found abundantly in telomeric DNA, is particularly susceptible to oxidative damage, leading to telomere shortening and strand breakage [24]. Chronic inflammation can trigger the release of pro-inflammatory cytokines such as TNF-α and IL-6, which induce oxidative stress and support cell growth [25]. The activation of signaling pathways like NF-κB by these cytokines can lead to increased ROS production and telomere damage. 

When telomeres reach a critically short length, they trigger the activation of repair proteins such as ATM and ATR [14,26]. Repeated damage response can result in cellular aging or apoptosis. While telomeres form protective T-loops to shield themselves, any damage or disruption to these structures can lead to further shortening. Epigenetic modifications, such as DNA methylation, can influence telomere length and telomerase function [27]. For instance, methylation of the TERT gene’s promoter region can constrain telomerase activity. The sheltering complex comprises essential proteins such as POT1, TRF1 and TRF2, which play a crucial role in protecting telomeres from being identified as DNA breaks [28]. Dysregulation of these proteins can lead to accelerated shortening and disruption of telomeres. Additionally, damage to DNA, specifically extremely short telomeres, can trigger the activation of the tumor suppressor protein p53, resulting in senescence, apoptosis or cell cycle arrest [29]. Persistent p53 activation due to chronic telomere shortening has been linked to tissue deterioration and aging. Inflammation and stress induce cellular reactions involving the AP-1 transcription factor and mitogen-activated protein kinase (MAPK) signaling pathways, potentially leading to increased ROS generation and telomere degradation [30]. Moreover, telomere length plays a crucial role in affecting the process of autophagy, essential for cell maintenance [31]. If a cell has short telomeres, it may undergo HIV and parasitic infections that can result in oxidative stress and inflammation [4,32].

HIV infection is linked to immunological activation and chronic inflammation, which causes immune cells’ telomeres to shorten and their production of ROS to increase [33]. Infections like HIV can affect telomere shortening through various routes, including enzymatic and signaling pathways. Increased production of pro-inflammatory cytokines and reactive oxygen species (ROS) can occur during infection when the nuclear factor kappa-light-chain-enhancer of activated B cells (NF-κB) pathway is activated [34]. Consequently, ROS cause oxidative damage to DNA, including telomeres. Another signaling route triggered during infections that may result in elevated ROS generation is the mitogen-activated protein kinase (MAPK) pathway [35]. ROS produced by the immune system can directly harm telomere-associated DNA [23]. Due to their high guanine concentration, repeated DNA regions known as telomeres are especially vulnerable to oxidative damage [23]. The chromosome-ending enzyme telomerase, responsible for appending telomeric repeats, can become dysfunctional due to prolonged exposure to oxidative stress [36]. As a result, telomere maintenance and repair are unsuccessful.

During HIV-1 infection, pro-inflammatory Th1 cytokines (IL-2, IFN-γ, TNF-α) are often upregulated while anti-inflammatory Th2 cytokines (IL-4, IL-10) are normally downregulated [37]. These alterations are a reflection of the complex interactions that affect immunological dysfunction and disease progression between the virus and the host immune system. Immunological senescence is characterized by low numbers of naïve T cells, higher frequencies of differentiated CD28^−^CD57^+^ T cells with reduced proliferative ability, a reduced CD4/CD8 ratio, oligoclonal expansion of CD8 T cells and gradual shortening of telomeres [38]. It has been reported that HIV infection accelerates the shortening of the telomere length in an individual, thus leading to biological aging [39,40,41,42,43,44,45].

Cytokines such as IL-4, IL-5, IL-9, IL-10 and IL-13 have a role in regulating immunological responses during parasitic infections [46,47]. They play a role in downregulating the immunological inflammatory response, attracting and activating immune cells, promoting the development of the immunoglobulin E response and eliminating parasites [46]. Various immune cells, including mast cells, eosinophils and T-helper cells, produce these cytokines. The type 2 (Th2) immune response, which includes B-cell-produced antibodies like IgA and IgE plus cytokines like IL4, is the hallmark of the immunological response to helminths such as *Ascaris lumbricoides*, *Trichuris trichiura*, and the hookworms *Necator americanus* and *Ancylostoma duodenale* [48]. Pro-inflammatory cytokines such as interferon-gamma (IFN-γ), interleukin-12 (IL-12), and tumor necrosis factor-alpha (TNF-α), are produced in large quantities in response to protozoan parasites (*Plasmodium* spp., *Trypanosoma* spp., *Entamoeba* spp. and *Toxoplasma gondii*) and are essential for the activation of macrophages and the destruction of intracellular parasites [49]. Furthermore, CD4^+^ T cells play a major role in the immune response to protozoan parasites through the release of cytokines that activate macrophages and promote parasite elimination [50]. Inflammatory diseases and parasites can produce more reactive oxygen species (ROS), which may increase the rate of telomere attrition [51]. It was indeed found that parasitic infections lead to telomere length shortening and accelerated biological aging [51,52,53,54,55,56,57].

Parasite infections have been suggested to influence the pathogenesis of HIV-1 infection because they have modulatory effects on the human immune system [58]. As a result, those who are afflicted are proposed to be more susceptible to HIV-1 infection and less able to handle it [58]. Chronic parasitic infection may inhibit the immune system’s defenses against HIV-1, while concomitant immunological activation may cause HIV-1-infected individuals to lose CD4 cells more quickly [59]. Furthermore, HIV-1-specific CD4^+^ and CD8^+^ proliferation and cytokine production may be suppressed by immunoregulation in response to parasite infection, which may impair control of HIV-1 replication and may enhance cellular vulnerability to HIV-1 infection as well [60]. Malnutrition and anemia, also associated with telomere shortening, were reported in individuals coinfected with HIV and parasites [61].

In contrast to how parasites interact with their hosts, HIV negatively impacts the host by impairing immune functions. With respect to human disease, parasitic-mediated immune regulation may have both advantageous and disadvantageous effects [62]. 

Enzymes, structural proteins, transport proteins, signaling proteins, receptors, chaperones, ion channels and apoptotic proteins are essential for immune response and cellular function. Their changes during infections can substantially affect the course of a disease. HIV and parasites frequently target or modify enzymes, which are crucial catalysts in metabolic pathways that enable their survival and replication [63]. For example, HIV protease breaks down viral polyproteins into useful proteins required for virus assembly. Similarly, parasite enzymes such as proteases facilitate the invasion of host tissue and the uptake of nutrients [64]. Maintaining the integrity and functionality of cells depends on structural proteins, which include those that make up the extracellular matrix and cytoskeleton. HIV can alter cytoskeletal proteins, limiting immune cell movement and promoting cellular dysfunction [65]. The extracellular matrix might change due to parasites. Chaperones support the folding and stability of proteins, which is essential for preserving cellular function under stress [66]. To guarantee that viral or parasitic proteins fold correctly and support the creation and maintenance of infections, HIV and parasites may take over host chaperones. Apoptotic proteins control the process of programmed cell death, which is essential for tissue homeostasis and immunological responses [67]. To extend host cell survival and promote viral replication or parasite survival within host tissues, HIV and parasites can interfere with apoptotic pathways [68].

ART effectively controls active viral infection by reducing HIV replication in plasma to undetectable levels [69]. Nevertheless, it fails to eliminate latent HIV reservoirs, which means that chronic immune activation and ongoing low-level viral activity exist [69]. HIV-positive people may have persistent immunological dysfunction despite viral suppression, characterized by dysregulated immune signaling pathways and altered cytokine profiles [70]. These factors can impact immune surveillance and responses against opportunistic diseases, such as parasites. Although antiretroviral therapy (ART) is an effective means of inhibiting active HIV replication and enhancing immune function in those under treatment, HIV latency, telomere shortening, immune dysregulation, changed cytokine profiles and signaling pathway activation all contribute to persistent immunological deficits and heightened vulnerability to parasitic infections [71].

There are limited studies on the association between telomere length and parasite single infection. In addition, although there are no reported studies on the association between telomere length and HIV and parasite coinfection, data from studies investigating the association between telomere length and HIV [39,41,42,43,44,45,71,72,73,74,75,76,77,78,79,80,81,82,83,84,85,86,87,88,89,90,91,92,93,94,95,96,97,98,99,100] and parasite [51,52,53,54,55,56,57] single infections support the hypothesis that HIV and parasite coinfection may further accelerate telomere length shortening and biological aging. Furthermore, investigating the connection between telomere length and HIV–parasite coinfection is crucial to determine whether biological aging can further be accelerated by parasites and viral infections, which will also aid in the development of efficient treatments and vaccines. This systematic review aims to summarize the effects of HIV infections on telomere length, helminth infections on telomere length and HIV–helminth coinfections on telomere length. However, articles on the latter (dual infection), which is the focus of our work, could not be found upon searching the literature during the study period of 1990–2024.

## 2. Method

This systematic review compiled relevant information from published studies investigating whether HIV and parasite single infection and coinfection influence telomere length shortening. A narrative approach was used to review relevant and available data on this topic. This systematic review was registered under PROSPERO (reference: CRD42024535337).

### 2.1. Search Strategy 

Google Scholar, Web of Science, Scopus, Science Direct and PubMed were used to search for relevant studies using the following keywords: “Telomere length and Human Immunodeficiency Virus/HIV infection”, “telomere length and parasite/parasitic infection” and “telomere length and Human Immunodeficiency Virus/HIV and parasite/ parasitic infection”. The Preferred Reporting Items for Systematic Reviews and Meta-Analyses (PRISMA) 2020 guidelines were followed to analyze and report the relevant studies. Articles published in the English language from January 1990 to May 2024 were included.

### 2.2. Study Selection, Study Quality, and Data Extraction

To identify appropriate literature based on tittles, abstracts and full texts, exclusion and inclusion criteria were followed by the main author (E.D.M). The eligibility of the literature was verified and accepted by the co-authors (J.M., P.N. and Z.L.M.-K.) The quality of the relevant data retrieved from the literature was divided into four categories: high, moderate, poor and extremely low [101]. This was evaluated using the grading of recommendations, assessment, development, and evaluations (GRADE) system [102].

### 2.3. Inclusion Criteria 

The inclusion criteria were as follows:

Articles published in January 1990–May 2024.

Published in English. 

Human and animal studies. 

Cohort, case-controlled studies and cross-sectional studies.

Articles reporting on telomere length and HIV infection.

Articles reporting on telomere length and parasite infection.

Articles reporting on telomere length and HIV and parasite coinfection.

### 2.4. Exclusion Criteria 

The exclusion criteria were as follows:

Articles published prior to January 1990.

Reviews, research letters, conference abstracts, book chapters and editorial corrections.

Articles not published in English.

Studies including major chronic infections and diseases. 

**Figure 1:**
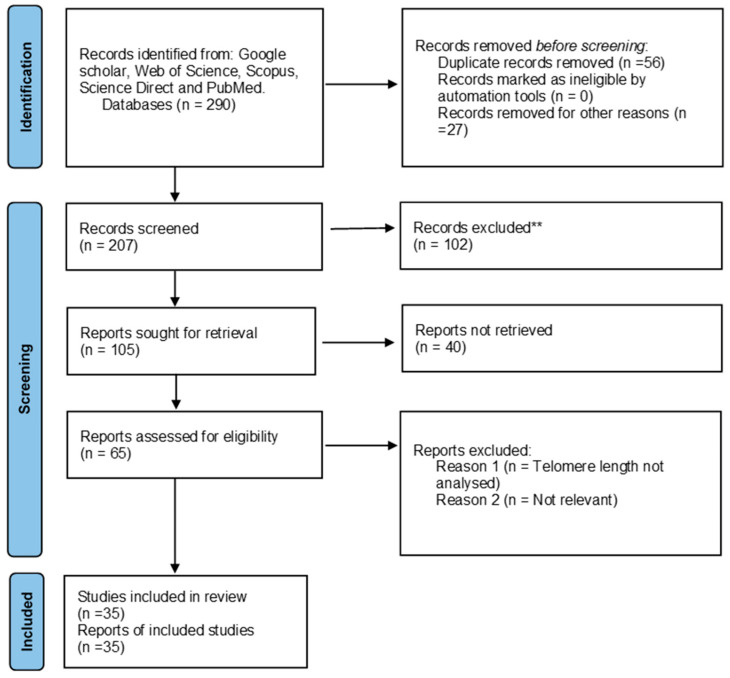
PRISMA flow diagram showing the article selection process used to collect, screen and identify eligible data of relevant articles based on telomere length and HIV. ** Not related to the study.

**Figure 2:**
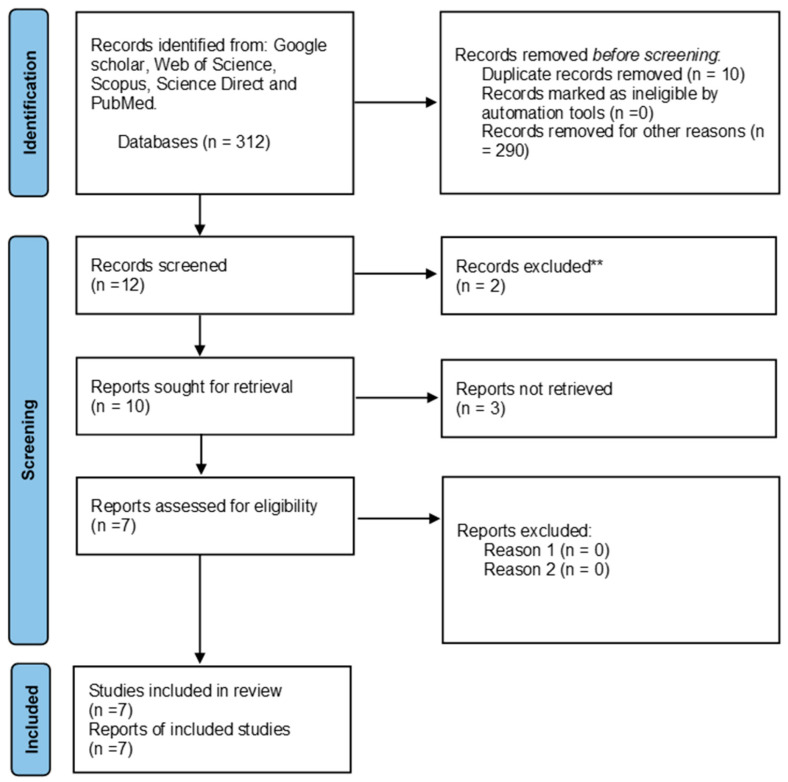
PRISMA flow diagram showing the article selection process used to collect, screen and identify eligible data of relevant articles based on telomere length and parasites. ** Not related to the study.

The search criteria yielded a total of 290 articles for telomere length and HIV and 312 articles for telomere length and parasites in the previously mentioned databases. Of the 290 articles, 234 were retrieved after removing duplicated studies and further evaluated based on the eligibility criteria. Of the 65 reports assessed for eligibility, only 35 reports were included in the review that summarizes data found on the association between HIV and telomere length. Additionally, of the 312 articles, 302 were retrieved after removing duplicated studies and further evaluated based on the eligibility criteria. Of the 10 reports assessed for eligibility, only 7 were included in the review that summarizes data found on the association between HIV and telomere length. There were no studies reporting the association between telomere length and HIV and parasite coinfection. A PRISMA 2020 flow diagram was used to record the whole selection process (Figure 1 and Figure 2). 

## 3. Results

Table 1 summarizes all studies investigating the association between telomere length and HIV infection. Table 2 summarizes all studies investigating the association between telomere length and parasite infections. 

There were no published studies investigating whether HIV and parasite coinfection influence telomere length shortening. 

## 4. Discussion

This is the first systematic review to investigate the association between HIV and parasite single infection and coinfection and telomere length. Shorter telomere length was associated with HIV single infection [41,42,43,44,45,71,72,73,74,75,76,77,78,79,80,81,82,84,85,86,87,88,89,90,91,92,93,94,95,96,97,98,99,100] and parasite single infection [51,52,53,54,55,56,57]. No studies report on the association between HIV and parasite coinfection and telomere length.

Several studies reported telomere length shortening in HIV-infected humans [41,42,43,44,71,80,81,83,87,90,91]. These studies also support the idea that biological aging in HIV-infected individuals can be accelerated by telomere attrition, chronic inflammatory environment, immune activation, mitochondrial dysfunction and altered epigenetic patterns. HIV infection results in chronic immune activation, oxidative stress and inflammation [81]. Premature telomere length shortening after HIV infection may be caused by a number of variables, including oxidative stress, HIV viral Tat proteins, persistent immunological activation, inflammation and combination antiretroviral therapy (cART) [80]. HIV infection was associated with shorter LTL [73]. Several studies assessed the association between HIV infection and CD4^+^ and CD8^+^ T cells; they observed telomere shortening in CD4^+^ and CD8^+^ during HIV infection in participants [88,92,96,97,99]. Chronic immune activation continues even in HIV-positive patients whose antiretroviral medication effectively suppresses viral replication [106].

Nucleoside reverse transcriptase inhibitors in antiretroviral therapy may also cause accelerated telomere shortening as they inhibit telomerase activity [107]. Adults infected with HIV had shorter telomeres, increased levels of immunological activation, increased regulatory T cells, and increased CD4^+^ cells that expressed PD-1, even after receiving ART [89]. At clinically achievable concentrations, tenofovir (TDF) was discovered to be the most powerful telomerase inhibitor and to cause the greatest amount of telomere shortening [93]. Furthermore, individuals treated with tenofovir for four years showed reduced telomerase in CD4^+^ cells and lower telomere length in CD8^+^ cells [100]. Individuals receiving combination antiretroviral therapy (cART) with efavirenz had significantly shorter telomeres than those receiving nevirapine [74]. Delaying the start of antiretroviral therapy (ART) in HIV-positive individuals, even by a few weeks, was associated with significant and persistent TL shortening [76]. On the contrary, the study presented in [78] reported that introducing ART in an HIV-infected individual limits the amount of the viral reservoir and stops telomere shortening and premature immune senescence. An increase in telomere length was observed in HIV participants who are on ART [75]. Studies [42,45,88,95] that found no correlation between ART and telomere length indicate that HIV-1 itself, rather than exposure to ART, is the source of rapid telomere shortening.

The relative telomere length of newborn leukocytes was reported to be influenced by maternal HIV infection or antiretroviral therapy exposure [72]. Regardless of the prophylactic treatment, telomere shortening was noted in 44.3% of children exposed to HIV in utero, but not infected with the virus [77]. HIV-infected children and adolescents had shorter telomeres in peripheral blood cells than age-matched HIV-uninfected participants [87,88]. Furthermore, among the children infected with HIV, the telomeres of the viremic children were shorter than those of the aviremic children [88,95]. The fact that children who test positive for HIV-1 have higher percentages of both activated and senescent CD8 cells, which inversely correlate with telomere length, provides additional evidence for the association between immunosenescence and biological aging [78].

Despite a lack of research and documentation regarding the relationship between the parasites and telomere length in infected humans, studies on parasite and telomere length in animals show that parasite infections might have an impact on telomere length shortening. A study on blue tits (*Cyanistes caeruleus*), discovered that chronic infection with haemosporidians that cause avian malaria and malaria-like illness was related to shorter telomeres in females compared to males [53]. Another study discovered that chronic malaria infection increased telomere shortening and shortened lifespan in mice [56]. According to [52], very low parasite exposure causes inflammation and oxidative stress, which accelerate telomere shortening and cellular senescence. According to [54], blood and tissue samples from the liver, lungs, spleen, heart, kidney, and brain of birds showed concurrent telomere shortening due to malaria infection. The study also found that compared to the control group, the blood of experimentally infected birds showed faster telomere attrition after infection. Similar findings were observed in [55], which discovered that human telomere shortening is caused by malaria infection. Haemosporidian *Leucocytozoon* parasitic infection was observed in tawny owls; infected owls had shorter telomere lengths than uninfected owls [57]. The relationship between telomere length and parasite (*Tetracapsuloides bryosalmonae*) load was investigated in wild brown trout (*Salmo trutta*) [24]. The *Tetracapsuloides bryosalmonae* parasite is known to cause proliferative kidney disease (PKD). PKD symptoms and the parasite load were linked to growth delays, and no significant associations between parasite burden and telomere length or antioxidant (AO) levels were discovered. Furthermore, these findings indicated that the various elements of the AO responses are linked to kidney hyperplasia and development, and that telomere length may represent quality in terms of an individual’s capacity to grow despite the parasite infection. Findings from the above-mentioned studies suggest that parasite infection can accelerate telomere length shortening in both humans and animals.

Since there are no studies investigating the association between HIV and parasite coinfection and telomere length, the findings above thus support our hypothesis that HIV and parasite coinfection indeed further accelerates telomere shortening. Future research should concentrate more on the relationship between telomere length and soil-transmitted helminth infections, human parasite infections, and the effects of parasite abundance on host telomere length.

## 5. Limitations

The studies on the association of parasites and telomere length were more based on malaria infection and animals, rather than humans, which might weaken the strength of our findings since different parasites affect different hosts in different ways. Th1 and Th2 cells, which are the most important factor in HIV and helminth coinfection, were not reported in the included studies. More reported studies were conducted in European countries, so regions like sub-Saharan Africa that have high HIV and parasite prevalence were less studied. The current study reported only articles that were written in English. There are no studies reporting the association between HIV and parasite coinfection and telomere length. There was a size imbalance between the studied groups [43,44,45,78,82,88,89,91,92,94,95,96]. Confounders such as sociodemographic factors and biochemical and full blood count parameters were not adjusted in some of the reported studies [43]. The required information on telomere length dynamics can be obtained by long-term studies, which also help validate or disprove the idea that early environmental factors influence an adult’s susceptibility to prevalent diseases. Some studies did not compare HIV-infected and uninfected individuals [74]. In regard to assessing the association of different types of ART in HIV-infected individuals, the study periods should be years longer, since ART takes more time to influence telomerase and telomere length [93]. Guidance on how to promote better aging in HIV-positive patients can be obtained by understanding these mechanisms.

## 6. Conclusions

The present study found that HIV and parasite single infections accelerate telomere length shortening. From the data retrieved in the reported studies, we can hypothesize that HIV and parasite coinfection accelerates telomere length shortening. HIV and parasite coinfections negatively impact the host immune system, and, paying attention to the documentation or studies conducted, these infections receive less attention. More studies should be conducted on the association of telomere length and HIV and helminth coinfections and other coinfections. Additionally, how the treatments used for these infections affect telomerase and telomere length should be studied as well. These studies can help in producing new suitable treatments for chronic infections and biological aging.

Furthermore, there is a large gap in genomics. Human genomes and infections, genome integrity caused by infections and coinfections, telomere length, and coinfections, mainly HIV and helminths, should be given more attention. Since some species of helminths result in cancer, further studies should focus on the association between cancer, helminths, HIV, and other infections, as well as the shortening of the telomere length.

In conclusion, prevention of HIV–helminth coinfection should be our priority, followed by educating the citizens about these kinds of coinfections and how to prevent them. Citizens should also be encouraged to take regular tests, as many citizens are unknowingly infected. To save money and state resources on vaccines for coinfections, supplements or treatments for healthy telomerase should be created.

## Figures and Tables

**Table 1:** Studies associating HIV infection with telomere length.

Aims of the Study	Methodology	Overall Findings	Reference
To examine the relative length of telomeric duplicates in infants with unrecognized perinatal HIV infection.	In total, 62 mothers infected with HIV and 80 healthy newborns (control group) were used in a cross-sectional study. The main group was subdivided into 2: (n = 41): newborns whose mothers had a detectable viral load prior to delivery. (n = 21): newborns whose mothers’ viral loads were undetectable. DNA was extracted from whole venous blood samples and analyzed.	The relative telomere lengths in newborns of HIV-infected mothers were significantly lower (0.69 (0.66; 0.72)) than in newborns of the control group (1.1 (0.97; 1.22)) (*p* < 0.001). There were no significant changes in the relative length of telomeres between neonates of mothers who had a viral load at the time of birth and those who did not: 0.69 (0.66; 0.73) and 0.69 (0.63; 0.72).	[72]
To investigate the relationship between leukocyte telomere length (LTL) and anti-Müllerian hormone (AMH) in order to better describe the relative contribution of clinical and sociodemographic variables to ovarian aging in women with HIV (WWH).	Women with HIV (WWH) and HIV-negative women aged 12–50 years were included in the study. LTL and AMH levels were determined using qPCR and ELISA, respectively. Women were studied during their peak reproductive (<35 years) and late reproductive (≥35 years) life stages. Parameters associated with AMH and ΔAMH/year were analyzed using multivariable mixed-effect linear or logistic regressions while adjusting for significant confounders.	Despite being of comparable age, WWH had shorter LTL and lower AMH levels than HIV-negative controls. After adjusting for other variables, HIV was linked with 20% lower AMH levels in women under the age of 35, while shorter LTL was associated with AMH levels less than 2 ng/mL in women over the age of 35. ΔAMH/year was related to initial AMH level in older women and to age in younger women throughout time.	[73]
To evaluate the influence of specific HIV-related variables on patients’ relative telomere length (RTL) and to compare patients’ RTL within and between different cART classes.	In total, 176 male Caucasian HIV patients aged 18 or older who were receiving regular monitoring and treatment were included in a cross-sectional study. Real-time polymerase chain reaction (PCR) was used to confirm HIV infection. Blood samples (4 mL) were taken from each patient using conventional venipuncture techniques, placed in K2EDTA and serum tubes, and inactivated in accordance with standard procedure. By using the salting out method, DNA molecules were extracted from mononuclear cells, and their quality and concentration were assessed using the spectrophotometric method. The quantitative PCR technique (qPCR) was used to determine relative telomere length (RTL). Data were analyzed.	RTL mean was 2.50 ± 1.87. When two nucleoside reverse transcriptase inhibitors were used as the main treatment together with an integrase inhibitor, protease inhibitor or non-nucleoside reverse transcriptase inhibitor (NNRTI), there was no difference (*p* = 0.761) in RTL between the therapeutic groups. RTL may be significantly impacted by the period of HIV infection, CD4^+^ T-cell count and cART, including NNRTI. The length of HIV infection (*p* = 0.220) and the CD4^+^ T-cell count (*p* = 0.536) were not shown to be significant by Kendall’s correlation test. RTL was affected by cART with NNRTI (*p* = 0.018). Patients on cART who used efavirenz had telomeres that were noticeably shorter than those on nevirapine.	[74]
To examine the impact on blood telomere length (BTL) after a year of transitioning from maintaining a standard triple therapy (TT) with an anchor medication and a two-NRTI backbone to a dual therapy (DT) with dolutegravir + lamivudine.	Adults on sustained three-drug antiretroviral therapy (ART) who were virologically suppressed and either switched to DT at baseline (BL) or continued to use triple therapy (TT) were included in this longitudinal, prospective, matched, controlled trial. In terms of age, sex, years since HIV diagnosis, years on ART, and anchor medication, the dual therapy (DT) and TT groups were matched 1:1. Monochrome multiplex qPCR was used to evaluate blood telomere length (BTL) at baseline and 48 weeks later (W48). Data were analyzed.	The BTL averages for the two groups were similar at BL (*p* = 0.973). The viro-immunological status remained constant at W48, and the mean BTL increased overall, by +0.161 (95%CI, 0.054–0.268) (*p* = 0.004). The DT group (*p* = 0.003) but not the TT group (*p* = 0.656) had a significant mean BTL gain, according to the within-group analysis. Switching to dolutegravir + lamivudine was associated with a greater gain in BTL than continuing triple treatment after the first year in patients with virologically suppressed PLWH.	[75]
To determine if immunological reconstitution interference or telomerase inhibition is the cause of the adverse effects of tenofovir on telomere length (TL).	In total, 128 long-term aviremic HIV adults receiving treatment with either tenofovir-sparing (n = 49) or tenofovir-containing (n = 79) regimens were included. The TRAPeze RT Telomerase Detection Kit was used to measure telomerase activity in PBMCs, CD4^+^ cells and CD8^+^ T cells. TL was measured in whole blood, PBMCs, CD4^+^ T cells, and CD8^+^ T cells by quantitative PCR (qPCR). Flow cytometry was used to assess the distribution of T-cell maturation. Data were analyzed.	An adjusted analysis revealed that individuals receiving tenofovir treatment for at least four years had reduced telomerase activity in CD4^+^ (*p* = 0.012) and CD8^+^ T cells (*p* = 0.023), as well as shorter TL in CD8^+^ T cells (*p* = 0.04). Lower percentages of recent thymic emigrant (RTE) CD4^+^ cells (*p* = 0.031) and PD1 marker expression (*p* = 0.013) were also associated with tenofovir therapy.	[100]
To investigate the pace of change in epigenetic age in PLWH after HIV infection but before startingActive Antiretroviral Therapy (HAART).	Cryopreserved peripheral blood mononuclear cells from 101 HIV-positive men were used to isolate DNA; the baseline visit was less than two. Visit 1 was five years following HIV seroconversion; Visit 2 was a follow-up visit less than 1.5 years before HAART was started; and 100 HIV-uninfected men were matched based on age and visited at similar intervals. Five clocks were used to estimate DNA methylation (DNAm) age: Pan-tissue, Extrinsic, Phenotypic, Grim, and Skin and Blood age. Additionally, a DNAm-based estimate of telomere length (DNAmTL) was determined. The baseline parameters linked to the rate of aging, which is defined as (DNAm age visit 2–DNAm age visit 1) (age visit 2–age visit 1), were examined using multivariate linear regression models. Data were analyzed.	For the Pan-tissue clock (1.9 vs. 0.9 epigenetic years per year of chronologic age, Welch’s *t*-test *p* < 0.001), Extrinsic clock (2.1 vs. 0.9, *p* < 0.001), and Phenotypic clock (1.8 vs. 0.8, *p* < 0.001), the average rate of aging was significantly higher in HIV-infected males compared to uninfected men. The Skin and Blood clock showed a slight difference in the rate of aging between males with and without HIV infection (1.4 vs. 1.1 years per year of chronologic age, *p* = 0.052), whereas the Grim clock showed no significant difference in the rate of aging (0.83 vs. 0.75, *p* = 0.50). When compared to males who were not infected with HIV, the average rate-of-shortening in DNAmTL was approximately three times higher in HIV-infected men (−0.056 units per year of chronologic age vs. −0.019, *p* < 0.001).	[41]
To determine the influence of clinical and HIV-related factors, such as specific ARVs, on longitudinal TL dynamics.	QPCR was used to quantify the change in TL in peripheral blood mononuclear cells in 107 patients who had longitudinal samples available prior to and during suppressive ART. Age, sex and the CD4/CD8 ratio were among the uni- and multivariable estimates for longitudinal TL dynamics that were obtained using mixed-effects multilevel modeling. We evaluated the impact of two interventions on TL in 798 extra subjects from their previous longitudinal studies: (1) an individual TL-polygenic risk score ([TL-PRS] based on 239 single-nucleotide polymorphisms) and (2) individual antiretrovirals. Data were analyzed.	TL decreased considerably (median −2.12%/year; IQR, −3.48% to −0.76%/year; *p* = 0.002) with untreated human immunodeficiency virus (HIV) infection (median observation, 7.7; interquartile range [IQR], 4.7–11 years). There was no indication of either a TL rise or drop with suppressive ART (median observation, 9.8; IQR, 7.1–11.1 years; *p* = 0.329). While specific antiretrovirals did not affect TL change (all *p* > 0.15), the TL-PRS did (global *p* = 0.019).	[42]
To study the effect of HIV and methamphetamine (METH) on leukocyte telomere lengths (LTLs) and the relationships between LTL and other aging biomarkers.	Cross-sectional analysis of 161 people, divided into four groups based on their HIV and methamphetamine (METH) dependence status: HIV-METH− (n = 50), HIV-METH+ (n = 29), HIV + METH− (n = 40), and HIV + METH+ (n = 42). The relationships between leukocyte telomere length (telomere to single-copy gene [T/S] ratio) and demographic and clinical data, as well as a panel of biomarkers of inflammation and endothelial activation measured in blood and cerebrospinal fluid (CSF), were analyzed. Data were analyzed.	HIV and METH were independently associated with a shorter T/S ratio (R^2^ = 0.59, *p* < 0.0001). Shorter T/S ratios were also associated with higher levels of plasma C-reactive protein (*p* = 0.0036) and CSF VCAM-1 (*p* = 0.0080). A lower T/S ratio was associated with an increased risk of cardiovascular disease (*p* < 0.0001) and stroke (*p* < 0.0001), weaker motor functioning (*p* = 0 <037) and processing speed (*p* = 0 <023), more depressed symptoms (*p* = 0.013), and more CSF neurofilament-light (*p* = 0.003).	[43]
To assess any independent relationships between the initiation of ART and TL in individuals in the Zurich Primary HIV Infection Study (ZPHI) who have documented primary HIV infection (PHI), and to calculate the effect of starting ART early in comparison to other variables that are known to be associated with TL, like age.	Researchers used quantitative polymerase chain reaction to evaluate total lymphocyte leukemia (TL) in peripheral blood mononuclear cells from study participants who had samples accessible for at least six years. After obtaining univariable and multivariable values from mixed-effects models, researchers assessed the relationship between baseline and longitudinal TL and postponing or stopping ART. Data were analyzed.	The median ART delay was 25, 42 and 60 days, respectively, in the first (shortest), second and third (longest) ART delay tertiles, among 105 PHI participants (median age 36 years, 9% women). Longer baseline TL (P for trend = 0.034) and longer TL over a 6-year period were associated with the first ART delay tertile, but only when ART was continuous (*p* < 0.001) and not stopped for more than 12 months (*p* = 0.408). After adjusting for age, those in the second and third ART delay tertiles had 17.6% (5.4–29.7%; *p* = 0.004) and 21.5% (9.4–33.5%; *p* < 0.001) shorter TL in multivariable analysis, with little variation in effect according to clinical factors.	[76]
To assess the acute and long-term effects on telomere shortening of two ARV prophylaxes: lopinavir/ritonavir (LPV/r) and lamivudine (3TC).	The study examined the relationship between telomere shortening and health outcomes at six years of age in children who are HIV-exposed uninfected (CHEU). Using qPCR, telomere length in 198 CHEU at seven days of life, week 50 and year 6 was assessed. Data were analyzed.	Regardless of the preventive therapy, 44.3% of CHEU had telomere shortening at week 50. With the exception of motor abilities, telomere shortening was not linked to poor growth indicators or cognitive outcomes at year 6 (MABC test n = 127, β = −3.61, 95%CI: −7.08, −0.14; *p* = 0.04).	[77]
To research how the biological and immunological aging profile in PHIV is related to the HIV-1 reservoir.	In total, 40 (perinatal HIV) PHIV patients who had undetectable viremia for at least 5 years prior to the sampling date and who began antiretroviral therapy (ART) before the age of 2 were enrolled. Samples from 37 children with 13.8 median age were used. Through the use of qPCR, the amounts of T-cell receptor rearrangement excision circle (TREC), a sign of thymic output, on CD4 and CD8 cells, as well as HIV-1 DNA copies on CD4 cells and relative telomere length (a hallmark of cellular senescence), were measured. Using flow cytometry, the immunological profile was evaluated. Multivariable Poisson regression was used to examine relationships between HIV-1 reservoir, age at ART commencement and molecular and phenotypic markers.	The telomere shortening (incidence rate ratio [IRR] = 0.15 [0.13–0.17]), immunosenescence (CD28–CD57^+^, IRR = 1.23 [1.21–1.26]) and immunoactivation (CD38^+^ HLADR^+^, IRR = 7.29 [6.58–8.09]) of CD4 cells were all significantly (*p* < 0.001) correlated with a higher HIV-1 reservoir. The percentage of CD4 senescent cells (2.89 [1.95–6.31] vs. 1.02 [0.45–2.69, *p* = 0.047) and higher HIV-1 reservoir levels (552 [303–1001] vs. 89 [56–365] copies/106 CD4 cells, *p* = 0.003) were associated with late ART beginning (after 6 months of age). TREC levels in CD8 cells were considerably lower in late-treated PHIV (1128 [486–1671] vs. 2278 [1425–3314], *p* = 0.042) and inversely correlated with the HIV-1 reservoir (IRR = 0.77 [0.76–0.79]).	[78]
To investigate the association between leukocyte telomere length (LTL) and whole-blood mitochondrial DNA (WB mtDNA) content in a group of HIV-positive and HIV-negative girls and women, both cross-sectionally and longitudinally.	Cross-sectional studies included 312 WLWH and 300 HIV-negative patients in total. Longitudinal studies included 228 WLWH and 68 HIV-negative people. DNA was extracted from whole-blood samples. MMqPCR was used to determine the relative LTL and mtDNA content in duplicate. Data were analyzed.	Cross-sectionally, there is no relationship between the two markers. Age, ethnicity, HIV and tobacco smoking were all independently associated with shorter LTL (N = 612, R^2^ = 0.28) and lower WB mtDNA in multivariable models (N = 612, R^2^ = 0.10). Longitudinally, there was a strong negative association between ΔLTL/year and WB ΔmtDNA content/year (R^2^ = 0.13, *p* < 0.001). Increased rates of LTL attrition and WB mtDNA loss were seen when HIV viral control was not maintained between visits.	[79]
To assess biological and environmental determinants of telomere length and assess peripheral leukocytes in HIV+ and healthy people.	In total, 325 HIV-positive participants were recruited, and systolic blood pressure and substance abuse were evaluated as covariates in the analyses. Leukocyte telomere length (LTL) was measured using quantitative real-time PCR, to measure βmicrogloblin expression as described by [103]. MRI scans and white matter hyperintensity (WMH) quantification were performed in 184 HIV-infected participants by fluid-attenuated inversion recovery (FRAIR) imaging. Data were analyzed.	LTLs were substantially shorter in HIV-positive people than in HIV-negative people (*p* < 0.001). TL was not associated with the viral load before cART, current CD4 COUNT, CD4 nadir or length of cART treatment. WMH was found in 36 HIV+ people (18.9%). WMH was strongly correlated with older age (*p* < 0.0001, shorter LTL (*p* = 0.0006), hypertension (*p* = 0.0128) and plaque of the carotid artery (*p* = 0.0374), but not with plasma viral load before Cart, current CD4 count, CD nadir, or length or type of Cart exposure.	[80]
To examine the factors linked to the baseline blood telomere length of participants enrolled in this randomized, open-label trial that compared ritonavir-boosted darunavir (DRV/r) plus raltegravir (RAL) with DRV/r plus tenofovir disoproxil fumarate/emtricitabine (TDF/FTC) in adults HIV-positive patients naïve to antiretroviral therapy (ART).	In total, 201 people who were chosen at random and had access to stored samples participated in a cross-sectional study. Monochrome quantitative multiplex polymerase chain reaction (PCR) was used to quantify the baseline telomere length. To determine mean differences and 95% confidence intervals (CIs) for the relationship between baseline telomere length and baseline attributes, multivariable predictive linear regression was employed.	With a mean age of 39 years, 89% of the participants were male, 83.6% of the participants were Caucasian, and 93% of the participants contracted HIV through sexual transmission. The mean estimated time since HIV diagnosis was 2.1 years, and the mean HIV-1 RNA load was 4.7 log10 HIV-1 RNA copies/mL. The mean baseline and nadir CD4 counts were 324 and 301 cells/lL, respectively, and the mean CD4/CD8 ratio was 0.4. Longer telomeres have been associated with higher age (per 10 years) (*p* < 0.001), HIV-1 RNA > 100,000 copies/mL (*p* = 0.001), CD4 count < 200 cells/lL (*p* = 0.037), poorer CD4/CD8 ratio (*p* = 0.018), statin medication (*p* = 0.004) and current alcohol intake (*p* = 0.035) in the univariate analysis. Age (*p* < 0.001) and HIV RNA > 100,000 copies/mL (*p* = 0.054) were shown to be independently associated with shorter telomere length in the multivariable analysis.	[83]
To assess the degree of systemic inflammation and comprehend the risk of age-associated illnesses in PLHIV on long-term suppressive ART by using a wide range of biomarkers for inflammation and immunological activation.	Blood samples have been collected from HIV-negative healthy controls (HIVNC, n = 41), PLHIV on ART for more than five years (ART, n = 53), and therapy-naïve PLHIV (Pre-ART, n = 43). In total, 92 inflammatory markers, sCD14, sCD163 and telomere length were measured in the samples. A variety of statistical tests were used to compare the research groups. The connections were examined using multivariate linear regression.	Eleven inflammatory markers were observed to differ significantly (*p* < 0.05) between the groups, including 4E-BP1, ADA, CCL23, CD5, CD8A, CST5, MMP1, NT3, SLAMF1, TRAIL and TRANCE. HIV-1 positivity and telomere length were significantly inversely correlated, according to linear regression analysis (*p* < 0.0001). CXCL1 (*p* = 0.048) and TGF~α (*p* = 0.026) strongly correlate with longer telomeres in the ART group, while TGFα (*p* = 0.042) significantly correlates with shorter telomeres.	[82]
To assess blood TL changes in an adult ART-naïve population in a sub-study of the NEAT001/ANRS 143 clinical trial comparing ritonavir-boosted darunavir with raltegravir or tenofovir disoproxil fumarate/emtricitabine.	A randomized, 1:1, open-label, 96-week noninferiority trial called NEAT001/ANRS143 was carried out in 78 clinical sites throughout 15 European countries. In 201 randomly selected participants, quantitative polymerase chain reaction (qPCR) was used to measure and compare changes in whole-blood telomere length. Data were analyzed	Those on tenofovir disoproxil fumarate/emtricitabine experienced a statistically significant greater increase in telomere length at week 96 compared to those on raltegravir. Adjusted for baseline telomere length, the difference in mean telomere length change across groups (tenofovir disoproxil fumarate/emtricitabine minus raltegravir) from baseline to week 96 was 0.031 (*p* = 0.009). Age, gender, length of known HIV infection, CD4 (baseline/nadir), CD8 cells, CD4/CD8 ratio, HIV viral load (baseline/week 96), alcohol and cigarette intake, statins or hepatitis C did not significantly affect this difference.	[84]
To examine how abacavir and tenofovir disoproxil fumarate (TDF) affected the length of blood telomeres in persons with HIV infection who were undergoing sustained virological suppression.	The participants in the prospective cohort were HIV-positive individuals with suppressed virological replication. Tenofovir disoproxil fumarate (TDF)-exposed individuals were compared to those who had never been exposed to TDF in terms of whole-blood telomere length, which was assessed using quantitative multiplex polymerase chain reaction analysis.	For the entire group, the mean TL increased by 0.042 (95% CI, 0.004–0.079) (*p* = 0.030). Compared to the non-TDF group, the TDF group’s telomere length improvements were noticeably less. TDF exposure was not linked to an independent adverse effect in the study limited to patients receiving N(t)RTIs. In comparison to those receiving N(t)RTI-sparing regimens or lamivudine as the only nucleoside, persons treated with two nucleosides in the non-TDF group likewise experienced considerably lesser gains in telomere length.	[85]
To investigate novel biological variables associated with poor bone mineral density (BMD) in women living with HIV (WLWH).	This research compares data from a subgroup of study participants in the CARMA (Children and Women: Antiretrovirals and Markers of Aging) study cohort at the Oak Tree Clinic with data from an unselected local reference population (CaMos). Assessments of Areal Bone Mineral Density (BMD) were performed. On the same day that the blood used for LTL measurement was processed, 200 µL of stored plasma was subjected to hepatitis C virus (HCV) RNA testing. Statistical analysis.	Relationships between health-related traits, cART, cellular aging (as determined by leukocyte telomere length, or LTL) and BMD of WLWH were examined using linear regression analysis. In comparison to controls (n = 290), WLWH (n = 73; mean age 43 ± 9 years) had poorer BMD Z-scores at the lumbar spine (LS) (mean difference = −0.39, *p* < 0.001) and total hip (TH) (−0.29, *p* = 0.012). In comparison to controls (n = 167), WLWH between the ages of 50 and 60 (n = 17) showed lower Z-scores at the LS (*p* = 0.008) and TH (*p* = 0.027). LS BMD was substantially correlated with BMI (R^2^ = 0.06, *p* = 0.042) and LTL (R^2^ = 0.09, *p* = 0.009) among WLWH. In WLWH, spinal BMD was negatively impacted. Lower BMD was highly correlated with reduced LTL, which may be related to the pathophysiology of WLWH and early aging.	[86]
To evaluate two aging biomarkers, DNA methylation (DNAm) age and telomere length, in a cohort of children with HIV infection who received early treatment, and to compare these aging biomarkers to those of children who were HIV-exposed uninfected (HEU) and HIV-unexposed uninfected (HUU).	In total, 120 HIV-positive, 33 HEU and 25 HUU children participated in this cross-sectional cohort study. Multiplex quantitative PCR was utilized to measure the length of the telomere. The Illumina 450 K array was used to quantify DNAm, and the acceleration residual from regressing DNAm age on chronological age was used to determine DNAm age. Data analysis.	In comparison to HEU children and HIV-positive children, telomere length (ln[Kb/genome]) was shorter in HIV-infected children (4.14 ± 0.85 vs. 4.53 ± 0.79, *p* = 0.038) and HEU children (4.05 ± 0.74 vs. 4.53 ± 0.79, *p* = 0.023). In both unadjusted analysis and after adjusting for cell type proportions, the age acceleration residual based on DNAm levels did not differ between HIV-infected (−0.003 ± 2.95), HEU (0.038 ± 2.39) and HUU (0.18 ± 2.49) children.	[87]
To determine the possible onset of age acceleration in HIV and outline the major biological pathways that may be disrupted during the HIV seroconversion phase.	Using the peripheral blood of 31 intravenous drug users, researchers assessed changes in DNA methylation and telomere length. These individuals were tracked longitudinally, with blood samples taken before HIV infection (T1), just after HIV infection (T2; 1.9 ± 1 years from T1) and at a subsequent follow-up period (T3; 2.2 ± 1 years from T2). PCR techniques were used to measure the absolute telomere length. The Illumina Human Methylation450 platform was used to produce methylation profiles. The Horvath method was used to evaluate methylation aging.	Between T1 and T2, telomere length reduced considerably (227 ± 46 at T1 vs. 201 ± 48 kbp/genome at T2, *p* = 0.045); between T2 and T3, no alterations were seen (201 ± 48 at T2 vs. 186 ± 27 kbp/genome at T3, *p* = 0.244). Over the course of HIV infection, methylation aging as determined by the age acceleration residual increased (*p* = 0.035). At a *q*-value < 0.05, CpG sites belonging to PCBP2 and CSRNP1 showed differential methylation between T1 and T2.	[44]
To examine whether shortened telomere length was associated with HIV infection, tuberculosis diagnosis, and 2-month death in a single cohort of HIV-infected and HIV-uninfected adults hospitalized with pneumonia.	HIV-infected patients with lung TB and pneumocystis pneumonia (PCP) were recruited. The questionnaire assessed current, symptoms, cardiopulmonary comorbidities, lifestyle and second-hand smoke exposure. HIV testing was performed and CD4^+^ was measured. AFB smear microscopy, GeneXpert MTB/RIF tests, bronchoscopy, BAL fluid, and pneumocystis tests were used to screen patients for tuberculosis. After blood was collected, telomere length was measured using Cawthon’s relative measurement of telomere length.	There were no significant demographic or clinical differences between HIV-infected and HIV-uninfected patients. Older age, men, cumulative pack-years smoked, alcohol use in the previous year and asthma were all associated with shorter telomere length (*p* = 0.21). HIV-positive participants exhibited substantially shorter telomeres than HIV-negative persons in multivariate analysis (β = −0.0621, 95% CI −0.113 to −0.011, *p* = 0.02). The length was not linked to tuberculosis or short-term mortality.	[81]
To investigate biological aging in connection to immunological activation and senescence markers in a perinatally HIV-infected kid cohort.	Real-time PCR was used to measure the length of the telomere and the levels of the excision circle of the T-cell receptor rearrangement in peripheral blood cells. Flow cytometry was used to examine CD4 and CD8 cells for differentiation, senescence and activation/exhaustion markers. Data were analyzed.	HIV had shorter telomere lengths than children with HEU and HUU children (*p* < 0.001 adjusted for age in general). HIV-naïve children had shorter telomere lengths than those on ART (*p* = 0.003 adjusted for age). HIVþ had lower T-cell receptor rearrangement excision circle levels and CD8 thymic emigrant cells (overall, *p* = 0.025 and *p* = 0.005). HIV-infected children have higher levels of senescent, activated and exhausted CD8 cells.	[88]
To investigate if T-cell activation in HIV-positive patients undergoing long-term antiretroviral treatment is associated with immunological senescence.	A total of 95 age-matched uninfected controls and 94 HIV-positive cases who were over 45 years old and on antiretroviral therapy (ART) had their immune phenotyping, thymic output and telomere length measured. Data were analyzed.	More immune activation (i.e., higher soluble CD14 [sCD14] level and higher percentages of CD38^+^ HLA-DR^+^ cells among both CD4^+^ and CD8^+^ T cells), more regulatory T cells, and a higher percentage of programmed cell death 1 (PD-1)-expressing cells among CD4^+^ T cells were present in cases, along with lower CD4^+^ T-cell counts and higher CD8^+^ T-cell counts. The percentages of CD57^+^ or CD27^−^CD28^−^ cells, or immune senescence levels, were similar in cases compared to controls. While peripheral blood mononuclear cells from cases showed shorter telomeres, they also contained more CD31^+^ naïve CD4^+^ T cells and single-joint T-cell receptor excision circle content.	[89]
To investigate the possible association between TL and immunological response 48 weeks after cART initiation and the function of OS and nitrosative stress in HIV-1 immunorecovery following cART.	After 48 weeks on sustained continuous antiretroviral therapy (cART), HIV-positive patients were evaluated to determine if their viral load had decreased to 50 copies per milliliter. Leukocyte TL was measured and divided into four groups. Using multivariate linear regression models, differences in mean increases were estimated according to tertiles of TL. Mean increases in CD4^+^ T cells after 48 weeks from cART beginning were also calculated.	The study included 122 patients, 86% of whom were male and 81% of whom were 50 years old when they started cART. Patients with long TLs had mean increases in CD4 that were higher than those with medium and short TLs (*p* = 0.007). Differences in mean CD4^+^ T-cell count increases according to TL remained statistically significant (*p* = 0.02) even after controlling for sex, age, CD4^+^ T-cell counts, viral load and hepatitis C infection at the start of cART. The outcomes were unchanged even after additional NOx and OS adjustments.	[90]
To assess the correlation between TL and immunological activation markers in a group of males who were either HIV-positive or not.	Men between the ages of 18 and 55 were previously recruited; 102 of them were HIV-positive and 41 were not. A minimum of three months of consistent antiretroviral therapy (ART) was mandated for patients with HIV infection. Assessment of immunologic parameters. Measurements were made of immune activation indicators and telomere length (TL). Data were analyzed.	Subjects with HIV had substantially shorter TL than those without HIV (*p* = 0.04). A substantial inverse connection between TL and sCD163, and consequently, monocyte/macrophage activation, was found in the HIV group by univariate analysis (ρ = −0.30, *p* = 0.003). After adjusting for age and smoking status, sCD163 (*p* = 0.05) and HIV-positive serostatus (*p* = 0.06) were found to be independent predictors of TL in multivariate modeling involving the entire cohort. Shorter TL in HIV is correlated with increased immunological activation.	[91]
To investigate LTL in the population infected with HIV and HCV and factors associated with a shorter LTL.	Children and women: Antiretrovirals and the Mechanism of Aging (CARMA) cohort research participants provided clinical data and blood. Factors identified as important in the univariate analysis were candidates for multivariate models.	Shorter LTL was associated with older age (*p* < 0.001), HIV infection (*p* = 0.04), active hepatitis C virus (HCV) infection (*p* = 0.02) and smoking (*p* < 0.003). Smoking was associated with shorter LTL only in HIV-uninfected subjects, indicating an interaction (*p* < 0.01). Age (*p* ≤ 0.002) and active HCV infection (*p* = 0.05) or maximum HIV RNA ≥ 100,000 copies/mL (*p* = 0.04) were associated with a shorter LTL in two models of HIV-infected persons, although other HIV diseases or treatment characteristics were not related.	[45]
To examine whether HIV infection and chronic stress associated with childhood trauma affect the length of the telomere and whether leukocyte TL (LTL) in particular is a risk factor for NCI.	Deoxyribonucleic acid (DNA) was extracted from each participant’s blood. Cawthon’s real-time quantitative PCR procedures were used to evaluate relative LTL.	LTL in HIV-positive people was considerably shorter than in HIV-negative people (F = 51.56, *p* =< 0.01).	[92]
To investigate the effects of the more widely used nucleoside reverse transcriptase inhibitors (NRTIs) on TL and telomerase activity in vitro in activated PBMCs and ex vivo in PBMCs from persons using NRTIs who were HIV-infected and HIV-uninfected.	QPCR was used to measure telomerase activity and telomere length (TL) in vitro in activated peripheral blood mononuclear cells (PBMCs) cultured with NRTIs and ex vivo in PBMCs from HIV-infected patients on NRTI-containing cART and from uninfected patients exposed to NRTIs.	In vitro, lamivudine, abacavir, zidovudine, emtricitabine and tenofovir substantially reduced the activity of telomerase in activated PBMCs. At a therapeutic dose of 0.3 μM, tenofovir was shown to be the most powerful inhibitor of telomerase activity and resulted in the highest shortening of TL in vitro. Compared to HIV-uninfected patients (n = 47; *p* = 0.011) and HIV-infected patients receiving non-NRTI-containing cART (n = 11; *p* < 0.001), PBMCs from HIV-infected patients receiving NRTI-containing cART (n = 39) exhibited significantly decreased telomerase activity. TL showed a significant adverse relationship with both age (*p* = 0.009) and the length of time on any NRTI (*p* = 0.01). The inhibition of telomerase activity by NRTIs, particularly tenofovir at therapeutic dosages, causes an accelerated shortening of telomere length in activated PBMCs.	[93]
To examine whether there is evidence that HIV-infected people have advanced biological aging relative to HIV-seronegative people by comparing the length of the telomere and CDKN2A expression.	In total, 236 HIV-infected persons over the age of 30 and 250 HIV-seronegative individuals were recruited from clinics in Cape Town’s township communities. Telomere length and CDKN2A expression in peripheral blood leukocytes were used to assess biological aging. Data were analyzed.	Telomere length was considerably shorter in HIV-infected people than in HIV-negative people (mean relative T/S ratio ± SE: 0.91 ± 0.007 vs. 1.07 ± 0.008, *p* < 0.0001). CD2NKA expression was higher in HIV-infected people than in HIV-negative people (mean expression: 0.45 ± 0.02 vs. 0.36 ± 0.03, *p* = 0.003). Socioeconomic characteristics were not linked to biological aging in HIV-infected people. However, biomarker values in patients on ART with undetectable viral load revealed increased biological aging in those with lower current CD4 cell counts.	[94]
To examine the potential effects of nucleoside reverse transcriptase inhibitor (NRTI) exposure during pregnancy or youth on leukocyte telomere length (LTL), a measure of cellular aging.	In total, 94 HIV+ children, 177 HIV-1-exposed uninfected (HEU) children who received antiretroviral therapy (ART) during pregnancy and 104 HIV-unexposed uninfected (HIV2) control children, all of whom were between the ages of 0 and 19, were included in the study. Relationships between explanatory variables and LTL were examined for all participants, HIV+/HEU children exclusively, and HIV+ children only using univariate and multivariate linear regression models.	When examining children of all ages collectively, there was no difference in LTL between the three groups after adjusting for gender and age. Male gender and older age were linked to shorter LTL in multivariate models. A detectable HIV viral load was also substantially linked to a shorter LTL for the HIV+ group alone (*p* = 0.007). In linear regression models, there were not any associations found between children’s LTL and their status as HIV-positive or exposed to prenatal ART.	[95]
To examine telomere length in blood cell populations as an indicator of replicative history in a large number of HIV patients.	Blood was collected using EDTA vacutainers, and within 4 h, peripheral blood mononuclear cells (PBMCs) were isolated on Ficoll–Hypaque gradients. Cell stimulation with phytohemagglutinin and cell cycle analysis. DNA isolation and terminal restriction fragment assays. Telomere repeat amplification was performed. Data analyses.	There is an inverse association between telomere length and the growth of immunosuppression, with HIV infection resulting in a five-fold or more acceleration of the aging of the immune system’s circulating PBMC component (*p* < 0.0001). Telomere lengths in 37-year-old patients were equivalent to those in uninfected 75-year-olds. Telomere loss was associated with AIDS progression and decreased proliferative potential of patient PBMCs but was unrelated to telomerase activity. The average telomere length was shorter in both CD4 and CD8 cells, with CD8 lymphocytes losing three times more telomeres.	[96]
To examine if the telomere length data may still permit high turnover rates in CD4^+^ T cells, which would deplete renewal and ultimately result in the depletion of CD4^+^ T cells.	Fresh blood samples were drawn for cross-sectional analysis from 12 HIV-positive individuals who were in the clinical latent stage. Frozen peripheral blood mononuclear cells (PBMCs) or newly isolated peripheral blood cells were used to produce purified CD4^+^ T cells. Telomeric restriction fragment length was determined. Using a mathematical model, the TRF data were interpreted in terms of CD4^+^ T-cell production.	The mean telomere lengths of CD4^+^ CD45RA^+^ and CD4^+^ CD45RO^+^ T cells from HIV-positive participants did not differ significantly from those of healthy controls (mean 6 SD for CD4^+^ CD45RA^+^ T cells was 8.6, 6, 1.1 and 9.0, 6, 0.9 kb, and for CD4^+^ CD45RO^+^ T cells, it was 7.4, 6, 0.8 and 7.5, 6, 0.9 kb, respectively). This model addresses earlier concerns by claiming that the virus’s division of CD4^+^ T-cell death is the reason for the normal TRF duration of CD4^+^ T cells during HIV-1 clinical latency. The average TRF duration of memory CD4^+^ T cells can only be extended by an enhanced priming rate of naïve CD4^+^ T cells to become memory cells. This could potentially counteract the shortening effect of increasing turnover in the CD4^+^ memory T-cell compartment.	[97]
To investigate TRF variations as a measure of T-cell replicative history and relate these to long-term variations in lymphocyte subpopulations after the start of prospective antiretroviral therapy.	Blood samples were collected from the participants, and ART protocol was followed. Density sedimentation was used to separate PBMC from whole blood, and magnetic beads were used to separate PBMC from CD3^+^, CD4^+^ or CD8^+^ T cells. The length of the terminal telomeric restriction fragment was analyzed. Telomerase assay was performed. The frequency of CD41 and CD81 peripheral blood T cells expressing HLA-DR, CD38, CD45RA, CD45RO, CD25, CD95 and CD28 was assessed using two- and three-color flow cytometry. Data were analyzed.	Following six to twelve months of effective antiretroviral medication, five of the seven patients showed increased T-cell mean TRF. After starting combination ART, the mean TRF of CD81 T cells increased in almost all of the patients. Over the course of the 48-week trial, CD81 T-cell median TRF length rose by 430 bp, and this increase was significant when compared to baseline (*p* = 0.007 by Student’s *t* test and *p* = 0.02 by Wilcoxon signed rank test). Compared to CD45RA^+^ naïve T cells, CD45RO^+^ T cells had shorter telomeres. The absolute number of CD4 cells expressing CD45RO (*p* = 0.004) or CD95 (*p* = 0.006) was higher than the substantial decline in CD8 cells expressing the CD45RO or CD95 antigens. The frequency of CD45RA^+^62L^+^ naïve cells (*p* = 0.003) and CD28^+^ cells (*p* < 0.001) changed in relation to changes in the mean TRF length in the CD4^+^ T-cell compartment. This suggests that the mean TRF length seen in the CD4 compartment may be related to new CD4 T-cell production after combination ART. The total number of naïve cells (*p* = 0.01) and CD4 cell telomere lengths were associated, but there was no association with the total number of CD28^+^ cells (*p* = 0.14).	[98]
To measure the length of the telomeres in peripheral blood mononuclear cells (PBMCs) from HIV-positive individuals in order to assess the effect of HIV infection on PBMC mitotic division and determine whether cellular senescence may be involved in immunological suppression.	In total, 13 seronegative donors with ages matching the HIV-1-infected patients’ (30–41 years old) were included, and PBMCs from them were separated by density gradient centrifugation using Ficoll–Hypaque. Telomere restriction fragment (TRF) length mean was determined. Data analyzed.	No significant difference was found between TRF lengths of control and HIV-infected patients with T4 lymphocytes/mm >2003 The Mandel–Whitney test revealed a significant difference in TRF lengths between PBMCs from HIV-infected patients with fewer than 200 T4 lymphocytes/mm^3^ and age-matched control donors (*p* = 0.0002) The telomere lengths of T4, T8 and B lymphocytes shorten in HIV-positive individuals with severe immunodeficiency.	[71]
To investigate whether HIV infection causes telomere length variations among T-cell subpopulations as a reflection of these cells’ history of replication and whether the remaining potential for T-cell replication is changed in infected individuals.	Peripheral blood T cells were isolated and fractionated. For the purpose of measuring telomere length, genomic DNA was extracted from purified peripheral blood CD4^+^ and CD8^+^ T cells and digested with HinfI and RsaI. PCR was used for telomere assay. Immobilized anti-CD3 and anti-CD28 were used to repeatedly activate CD4^+^ T cells. Data were analyzed.	Mean TRF length in CD4^+^ cells was significantly larger in the infected than in the uninfected twins (mean difference 1.2 ± 0.4 kb). HIV-positive twins’ TRF length in CD8^+^ cells is shorter than that of their uninfected twins’ (mean difference: 1.1, 6, 0.3 kb). In donors without HIV infection, TRF length in CD8^+^ T cells was found to be longer (mean difference 1.2, 6, 0.7 kb) than in CD4^+^ cells from the same individual; in HIV-positive twins, however, TRF length was longer in CD4^+^ cells (mean difference 0.9, 6, 0.4 kb). Ex vivo separated CD4^+^ or CD8^+^ cells from an independent panel of 15 (non-twin) HIV-positive donors examined or from HIV-negative controls showed low or no telomerase activity.	[99]

**Table 2:** Studies associating parasite infection with telomere length.

Aims of the Study	Methodology	Overall Findings	Reference
* To analyze the relationship between telomere length, cellular senescence, telomerase expression and aging-related activities during a single malaria infection.	In total, 16 healthy malaria-free Dutch people were recruited, and the CHMI Standard was followed according to [104]. Using an automated extraction technique, total nucleic acid was isolated from 481 blood samples from 16 study participants. As reported by [55], TL was measured using quantitative real-time PCR. RNA purification and cDNA purification were performed. The comparative CT method was used to estimate the relative gene expression of CDKNA2A, telomeres and antioxidants (GAPDH). TaqMan Gene Expression Assay was used to evaluate CDKNA2A expression, and the human hTERT TaqMan Assay was used to detect telomerase. The LEGEND plex Multi-Analyte Flow Assay kit was used to measure cytokine levels in Citrate Plasma at 3 time points: C−1, DT+1 and C+64. Blood tests were performed to monitor liver enzymes and renal function. Data were analyzed.	There was a significant change in the dynamics of TL in peripheral blood (Wald χ2 = 26.13, N = 139, *p* < 0.001). Infection with *P. falciparum* increased telomere shortening, with the shortest telomeres (Ime, *p* = 0.031) seen simultaneously with the highest parasite count (197,500 Pf/mL). Females had a mean TL of 13.79 kb (SD 3.67, 95% CI 13.41–14.17) compared to males who had a mean TL of 10.88 kb (SD 2.84, 95% CI 10.37–11.39, two-sample *t*-test t1, 261 = 9.01, *p* < 0.001). After controlling for age, gender and antimalarial therapy, telomere length and parasite density were found to be negatively correlated (Ime, *p* 0.006). Significant decrease in antioxidant gene expression with DT+1 compared to C−1 (all *p* < 0.05), indicating an altered oxidative–antioxidant balance during infection. There was a significant change in the kinetics of cellular aging markers between the day before infection (C−1) and the first day after treatment (DT+1), a decrease in TL (0.004) and increased CDKN2A expression levels on DT+1 (*p* < 0.001). Both markers were correlated with parasite density (*p* > 0.05). At C+64, TL and CDKN2A returned to baseline levels, with a significant difference in markers between C−1 and C−64 (all *p* > 0.05). Malaria parasitemia causes an oxidative stress response that shortens telomeres and causes cellular senescence.	[52]
To investigate the association between blood parasite infection (from the genera Plasmodium and Haemoproteus) and telomere length (TL) in a natural population of the blue tit (*Cyanistes caeruleus*).	The research was carried out in a wild population of nest-box breeding blue tits living in the southern section of Sweden’s Baltic Island of Gotland. The median longevity of adult birds observed in the population is 2 years, and the greatest lifespan reported throughout the study period is at least 6 years. DNA was extracted from whole-blood samples. QPCR was used to analyze the TL. The DNA extracted for TL analysis was examined for the presence of blood parasites (genera Haemoproteus and Plasmodium) to determine malaria status. Data were analyzed.	They discovered that chronological age had a considerable negative effect on TL. TL was shown to be significantly associated with malaria infection type; they discovered longer telomeres in those infected with Haemoproteus compared to Plasmodium, although this was only true in males. Telomere attrition rate (telomere shortening with age) was not associated with the type of parasitic infection (age infection type, F2,166.2 = 0.579; *p* = 0.562), so these associations were excluded in the final model. Furthermore, they discovered no association between infection severity and TL.	[53]
* To investigate the impact of a single acute Plasmodium falciparum malaria infection on the cellular aging dynamics of tourists.	The study included 38 people diagnosed with and treated for *Plasmodium falciparum* malaria at Karolinska University Hospital in Stockholm, Sweden. Field’s dyed tin and thick smears indicated 500–100,000 parasites per microliter of blood using conventional light microscopy. Automated hematology analyzers counted the counts manually according to the CLSI H20-A2 standard [105]. The QuantStudion 5qPCR instrument was used to quantify the telomere length and HBGI, HBGI 2 to quantify the single-copy gene. RNA was extracted from blood and converted to cDNA using SuperScript VILOTM. Telomerase expression was measured using the human TaqMan Copy Number Reference Assay, hTERT and GADPH Assay on a QuantStudio 5qPCR instrument. On a QuantStudio instrument, CDKN2A expression was evaluated using the TaqMan Gene Expression Assay. Data were analyzed.	When compared to 3 months after infection, the length was substantially longer after 12 months (mean diff = 0.39, 95% CI 0.58 to 0.21, N = 17, *p* < 0.001); however, there was no significant difference 12 months after infection compared to day 0 (mean diff = 0.07, 95% CI 0.12 to −0.26, N = 23, *p* = 0.460). Telomere dynamics were not affected by antimalarial treatment. Telomere (hTERT) expression was significantly reduced 10 days post-infection compared to day 0 (mean diff = −2.64, 95% CI 0.03 to −5.31, N = 7, *p* = 0.052). At 1 month post-infection, mean diff = 2.72, 95% CI 5.01 to 0.44, N = 6, *p* = 0.228. There was a substantial positive connection between telomerase expression and telomere length (r = 0.50, N = 39, *p* = 0.010). There was a significant negative association between CDKN2A expression and telomere length (r = −0.39, N = 39, *p* = 0.025), with a possible negative correlation between CDKN2A expression and telomerase activity (r = −0.30, N = 39, *p* = 0.062). The differential leukocyte counts were not significantly linked with telomere length.	[55]
To examine the possible functions of TL and antioxidant (AO) defense in *T. bryosalmonae*-infected fish, as well as their correlations with parasite burden and disease severity.	Young brown trout fish exposed to *T. bryosalmonae* were caught in a river. Using a salt extraction technique, genomic DNA for TL measurement was isolated from liver tissues. QPCR was used to measure TL. An EnVision plate reader was used for all AO defense measures, and the methods were modified to make them more appropriate for smaller sample volumes. Data were analyzed.	In comparison to fish that were less parasitized, the higher parasite load fish displayed more severe kidney hyperplasia, anemia and reduced body size. TL and parasite load did not exhibit a direct correlation. Shorter TLs were seen in smaller fish, which may indicate inferior individual quality. In comparison to more sensitive fish, the fish that were less sensitive to parasite-induced decreased growth measured as parasite load-adjusted fork length also had longer TLs.	[51]
To investigate the effect of blood parasitic hemorrhage on telomere dynamics in tawny owls.	Tawny owls in Southern Finland were studied and sampled according to their color. Telomere length and *Leucocytozoon* parasitemia estimation was performed using quantitative PCR. Data were analyzed.	Shorter telomeres were found in darker pheomelanic brown individuals compared to pale grey ones (color score: b = 20.01 ± 0.004, X_1_^2^ = 10:70, *p* = 0.001). While telomere length fluctuated over time based on coloring. There was a significant correlation between telomere length and age (X_2_^2^ = 0:86, *p* = 0.65), breeding experience (X_1_^2^ = 0:14, *p* = 0.70), sex (X_1_^2^ = 0:84, *p* = 0.36) or laying date (X_1_^2^ = 0:18, *p* = 0.66). The correlation between the frequency of infections and telomere length did not vary according to plumage colors (infection color score: X_1_^2^ = 0:35, *p* = 0.55), nor did the correlation change with age (infection x age class: X_2_^2^ = 3:19, *p* = 0.20).	[57]
To determine whether malaria infection causes parallel telomere shortening in blood and tissue samples from various avian organs.	Siskins (*Spinus spinus*) were infected by the avian malaria parasite *Plasmodium ashfordi*, and the telomere length in control and experimentally infected siskins was measured using quantitative real-time polymerase chain reaction (PCR). Data were analyzed.	When compared to control birds, the mean telomere length in blood cells from the experimental birds was 41% shorter at 105 DPI. Control birds showed no significant change in blood cell telomere length after 105 days (LME, F_1,152_ = 0.78, *p* = 0.38), with no effect of sex or the interaction sex ×DPI (all *p* > 0.63). Infected birds had shorter telomeres in all six major organs studied (liver, lungs, spleen, heart, kidney, and brain) compared to controls at 105 DPI (F_1,26_ = 21.9, *p* < 0.0001).	[54]
To determine the potential long-term effects of avian malaria in birds.	Great reed warblers (*Acrocephalus arundinaceus*) were infected with avian malaria, *Plasmodium* and *Haemoproteus* spp. DNA was extracted from the great reed warbler blood samples. For DNA-based detection and identification of malaria parasites, PCR was used, and for determining infection intensity, QPCR was used. Data were analyzed.	They observed that telomere length declined with age in great reed warblers (*p* < 0.0001), and more steeply in infected birds than in uninfected birds [as seen by the significant interaction age malaria infection status, *p* < 0.0001]. Malaria-infected birds had a considerably higher rate of telomere shortening than uninfected birds *p* < 0.0001. They investigated malaria infection status and telomere shortening in the birds’ first year of life; they found a difference between infected and uninfected individuals for all parasite lineages combined (*p* = 0.0001) and for the three most frequent parasite lineages separately. The severity of the infection appeared to be related to the degree of telomere shortening.	[56]

* Human studies. It is noteworthy that upon searching the databases, only two studies investigating parasite infection (malaria) and telomere length shortening were performed on humans.

## Data Availability

Not applicable.
